# A global perspective on coal-fired power plants and burden of lung cancer

**DOI:** 10.1186/s12940-019-0448-8

**Published:** 2019-01-28

**Authors:** Cheng-Kuan Lin, Ro-Ting Lin, Tom Chen, Corwin Zigler, Yaguang Wei, David C. Christiani

**Affiliations:** 1 Department of Environmental Health, Harvard T.H. Chan School of Public Health, 665 Huntington Avenue, Building 1, Room 1401, Boston, MA 02115 USA; 2 Department of Occupational Safety and Health, China Medical University, 91 Hsueh-Shih Road, Taichung, 40402 Taiwan; 3 Department of Biostatistics, Harvard T.H. Chan School of Public Health, 655 Huntington Avenue, Building 2, 4th Floor, Boston, MA 02115 USA; 4 Department of Epidemiology, Harvard T.H. Chan School of Public Health, 665 Huntington Avenue, Building 1, Room 1401, Boston, MA 02115 USA

**Keywords:** Coal capacity, Coal-fired power plants, Energy matrix, Environmental factor, Global burden disease, Lung cancer incidence, PM_2.5_

## Abstract

**Background:**

Exposure to ambient particulate matter generated from coal-fired power plants induces long-term health consequences. However, epidemiologic studies have not yet focused on attributing these health burdens specifically to energy consumption, impeding targeted intervention policies. We hypothesize that the generating capacity of coal-fired power plants may be associated with lung cancer incidence at the national level.

**Methods:**

Age- and sex-adjusted lung cancer incidence from every country with electrical plants using coal as primary energy supply were followed from 2000 to 2016. We applied a Poisson regression longitudinal model, fitted using generalized estimating equations, to estimate the association between lung cancer incidence and per capita coal capacity, adjusting for various behavioral and demographic determinants and lag periods.

**Results:**

The average coal capacity increased by 1.43 times from 16.01 gigawatts (GW) (2000~2004) to 22.82 GW (2010~2016). With 1 kW (KW) increase of coal capacity per person in a country, the relative risk of lung cancer increases by a factor of 59% (95% CI = 7.0%~ 135%) among males and 85% (95% CI = 22%~ 182%) among females. Based on the model, we estimate a total of 1.37 (range = 1.34 ~ 1.40) million standardized incident cases from lung cancer will be associated with coal-fired power plants in 2025.

**Conclusions:**

These analyses suggest an association between lung cancer incidence and increased reliance on coal for energy generation. Such data may be helpful in addressing a key policy question about the externality costs and estimates of the global disease burden from preventable lung cancer attributable to coal-fired power plants at the national level.

**Electronic supplementary material:**

The online version of this article (10.1186/s12940-019-0448-8) contains supplementary material, which is available to authorized users.

## Introduction

Coal-fired power plants are the dominant source of energy production, yielding > 40% of global electrical power since the 1970s [1]. Indeed, global production of coal increased nearly 2.2-fold from 1958 million tons of oil equivalent (Mtoe) in 1980 to 4270 Mtoe in 2010 [1]. However, air pollutants emitted from coal power plants and their potential impact on population health have aroused widespread concerns; fine particulate matter (PM_2.5_) can cause both short-term and long-term adverse health outcomes [2–4]. Long-term exposure to PM_2.5_ is associated with shorter life expectancy and higher mortality risks from lung cancer and cardiovascular diseases [5–8]. In fact, the International Agency for Research on Cancer (IARC) has listed several coal-fired power plant-related agents, including coal combustion, coal production, outdoor air pollution, and radon, as human carcinogens [9]. While lung cancer is prevalent, the proportion of cases attributed to environmental factors such as air pollution varies by country and is difficult to estimate [10]. Nonetheless, improved air quality has been correlated to better health [6, 11], prompting many countries to implement regulations on air pollution [12].

Most available estimates of health risk associated with electricity generation are oversimplified since they are calculated by multiplying a factor to air pollution levels (either PM_2.5_ or PM_10_) without considering the heterogeneous compositions of particles from different sources [13–15]. Moreover, lower global levels of PM_2.5_ are not necessarily associated with reduced adverse health effects, likely due to regional variations in composition [16, 17]. For example, satellite-driven PM_2.5_ measurement showed a high level of air pollution concentrated in sub-Saharan Africa [18]. Yet, a major component of that PM was dust from the earth’s crust rather than from human activities. Therefore, simply using PM to estimate health effects may result in misguided conclusions.

To clarify the long-term health effects from coal-fired power plants at the national level and linking the capacity market in energy economic to health externality, we aim to estimate changes in national lung cancer incidence decades after building or closing coal-fired power plants.

## Methods

### Study period and design

Annual lung cancer incidence rates from 2000 to 2016 among males and females from countries which have had coal-fired power plants were included in the analyses. Most countries in the study are located in Europe (38.55%) and Asia (27.71%) (Additional file 1: Table S1). Country names and geographical categories reflect the United Nations’ country classification [19].

### Dependent variables & independent variables

Annual lung cancer incidence rates were obtained from Global Burden of Disease Study [10]. Lung cancer codes were B101 or 162 in International Classification of Diseases version 9 (ICD-9); C028, 162, 231.1, or 231.2 in ICD-9CM; and C33, or C34 in ICD-10. Calculated age-adjusted incidence rates were based on the WHO 2000–2025 standard population for each country [20]. We use “independent variables” and “covariates” interchangeably throughout.

Electrical capacity of power plants that primarily relied on coal as generating fuel was the study of interest. *Coal capacity* was defined as the annual accumulation of generating capacity from every coal-fired power plant in a given country. Similarly, we define *plant capacity* as the accumulation of total generating capacity from all power plants in a country. *Non-coal capacity* was plant capacity minus coal capacity. *Coal percentage* was defined as the ratio of coal capacity to plant capacity for each country. Per capita *coal capacity* is the coal capacity divided by total population in the corresponding country. Total coal consumption is the annual coal usage in all sectors (including electricity, industrial and residential use, units in Quadrillion Btu) in a given country [21]. Capacity data was derived from the Utility Data Institute World Electric Power Plants Data Base [22]; we merged the WEPP database with incidence data by country and year. After matching, a total of 83 countries were included in the study.

We collected data on covariates of smoking prevalence, economic indexes, industrial indexes, and traffic indexes for each country. Annual smoking prevalence within each country was estimated, sex- and age-adjusted [23]. Per capita gross domestic product adjusted for purchasing power parity [GDP(PPP)] and inflation to base year 2011 USD was used to capture the country’s standard of living and healthcare level [24]. The indicator of CO_2_ emissions only from manufacturing industries and construction (% of total fuel combustion) was used to characterize industrialization [24]. Traffic index, or the level of urbanization, measured as the proportion of a country’s population living in urban areas, was applied to capture air pollutants emitted from all mechanical vehicles and public transports [24]. The missing data in North Korea and Taiwan were obtained from supplementary sources [25, 26].

### Data analysis

The longitudinal model for which we predict lung cancer incidence is the following Poisson regression:

$$ \log E\left[{\lambda}_{it}|{\boldsymbol{X}}_{\boldsymbol{it}}\right]={\upbeta}_0+{\upbeta}_1{\left[\mathrm{Per}\ \mathrm{capita}\ \mathrm{Coal}\ \mathrm{Capacity}\right]}_{\mathrm{i}\left(\mathrm{t}-\mathrm{T}\right)}+{\upbeta}_2{\left[\mathrm{Smoking}\ \mathrm{Prevalence}\right]}_{\mathrm{i}\left(\mathrm{t}-10\right)} $$

$$ +{\upbeta}_3{\left[\mathrm{Non}\ \mathrm{Coal}\ \mathrm{Capacity}\right]}_{\mathrm{i}\left(\mathrm{t}-10\right)}+{\upbeta}_4{\left[\mathrm{Traffic}\ \mathrm{Index}\right]}_{\mathrm{i}\left(\mathrm{t}-10\right)} $$

$$ +{\upbeta}_5{\left[\mathrm{Industrialization}\ \mathrm{Index}\right]}_{\mathrm{i}\left(\mathrm{t}-10\right)}+{\upbeta}_6{\left[\mathrm{Per}\ \mathrm{capita}\ \mathrm{GDP}\ \left(\mathrm{PPP}\right)\right]}_{\mathrm{i}\mathrm{t}} $$

$$ +{\upbeta}_7{\left[\mathrm{Total}\ \mathrm{Coal}\ \mathrm{Cunsumption}\right]}_{\mathrm{i}\left(\mathrm{t}-10\right)} $$

where index *i* denotes the country, *t* denotes the year, and *T* is the believed lag of per capita coal capacity before affecting the current lung cancer incidence rate *λ*_*it*_. For completeness, we consider three lags at *T* = 5, 10, 15 years for coal capacity and assume an adequate lag of 10 year for smoking [27] and other covariates, except for per capita GDP.

The model stated above is a marginal model; specifically, we are not concerned with how the effect varies across individual countries, but rather with the “overall” effect averaged over all countries. We must, however, account for this within-country variation across the years, for which generalized estimating equations (GEE) [28] is perfectly suited to handle. GEE’s strengths lie in its semiparametric properties: assuming no residual confounding or other sources of bias, GEE produces unbiased estimates of the beta coefficients, regardless of the within-country correlation structure specified, although a specification closer to the true correlation structure leads to lower standard errors.

The GEE fit was performed using the geepack package within R version 3.2.5 to estimate the effect of the selected covariates on standardized lung cancer incidence. We use an independence correlation structure, and fit for males and females separately, each weighted by the corresponding male and female populations. Figures were also drawn in R version 3.2.5.

### Falsification test

To investigate the possibility that general health improvements correlated with coal capacity may obscure our lung-cancer results, we identify colorectal and anal cancer (CRC) as falsification outcomes. Although one study reported the possible association between CRC mortality and NO_2_ [29], results from the other studies suggested negative or inconclusive association between PM and CRC [30, 31]. CRC was coded as B093, B094, 153 or 154 in ICD-9; and C18 to C21 in ICD-10 [10]. We applied the same models to CRC to examine any association with coal capacity.

### Burden of diseases analysis

We estimate the population attributable fraction (PAF) of lung cancer to coal-fired power plants in 2015 and predict the PAF in 2025 among studied countries. The PAF is the proportion of lung cancer incidence attributable to anthropogenic coal capacity. Detailed step-by-step calculations are summarized in the GBD study [10] and our previous work [32]. Briefly, to calculate *PAF*_*it*_, the PAF for country *i* in year *t*, we need the quantity *RR*_*it*_, the relative risk of lung cancer incidence given coal capacity at year *t* − 10, holding all other covariates, including smoking, fixed. This can be deduced immediately from our data analysis portion (10-year-lag model) using the relationship

$$ {RR}_{it}={RR}_0^{\mathrm{Per}\ \mathrm{capita}\ {\mathrm{coal}\ \mathrm{capacity}}_{\mathrm{i}\left(\mathrm{t}-10\right)}} $$

$$ {PAF}_{it}=\frac{P_{it-10}\times \left({RR}_{it}-1\right)}{1+{P}_{it-10}\times \left({RR}_{it}-1\right)} $$

where RR_0_ is the relative risk for every KW/capita unit increase in lag 10 coal capacity (1.585 for males, 1.851 for females) as we obtained from the 10 year-lag model (Table 2). *P*_*it* − 10_ is the proportion of males or females. *PAF*_*it*_ is useful, because we can then calculate the standardized attributable cases:

$$ \mathrm{Standardized}\ {\mathrm{attributable}\ \mathrm{cases}}_{it}={\mathrm{PAF}}_{it}\times {\mathrm{Population}}_{it}\times \mathrm{standardized}\ {\mathrm{incidence}\ \mathrm{rate}}_{it} $$

## Results

**Fig. 1:**
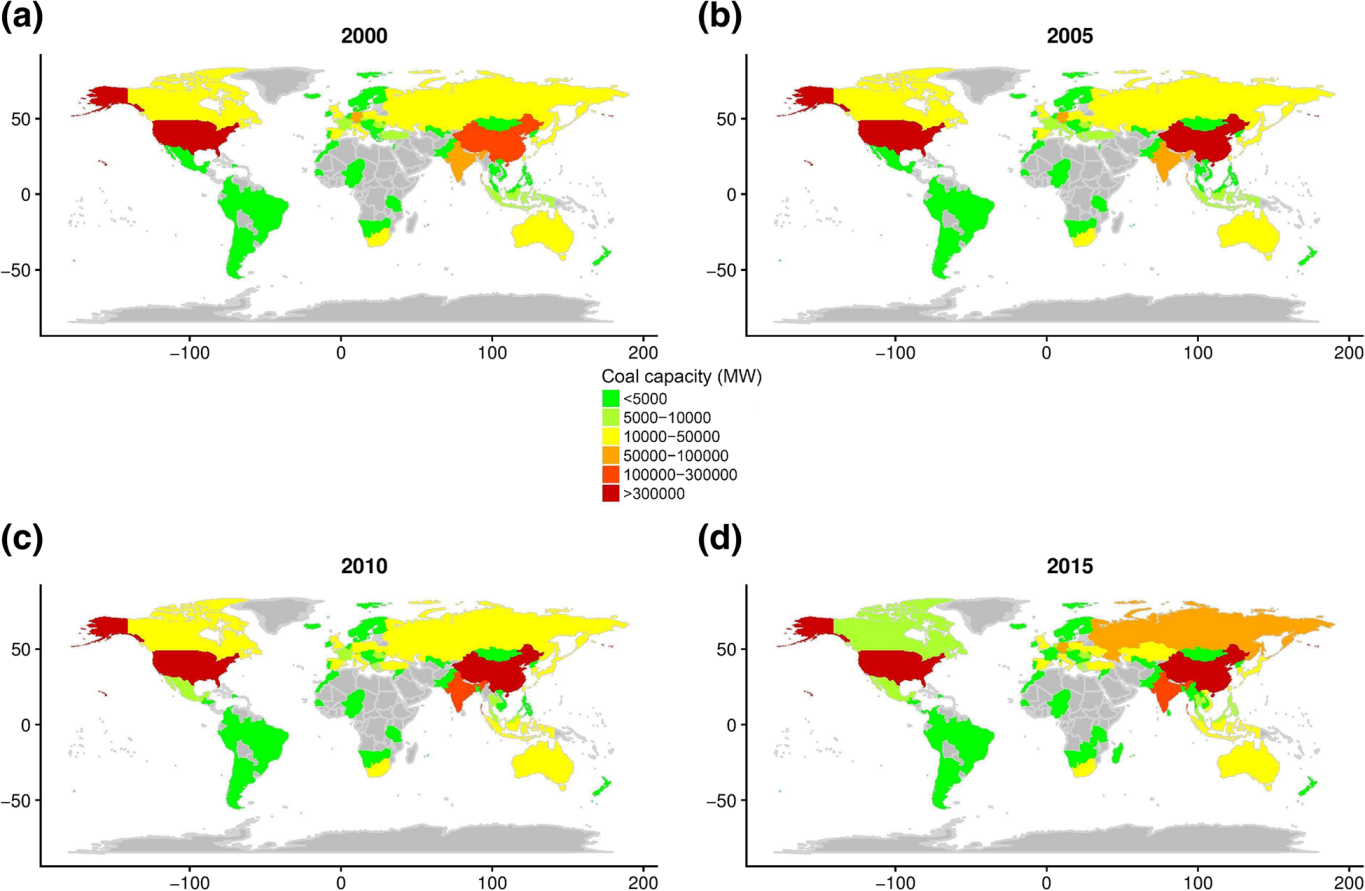
National coal capacity in (**a**) 2000; (**b**) 2005; (**c**) 2010 and (**d**) 2015

Coal capacities were calculated from a total of 13,581 generating units among 83 countries. All countries have complete 17-year follow-up data from 2000 to 2016. Coal capacities in four time points (years 2000, 2005, 2010, 2015) are mapped in Fig. 1. Coal capacity varied widely both within and between countries across time. Additional file 2: Figure S1 shows coal capacity, plant capacity, coal percentage and total coal consumption of the top 5 countries with the highest levels of coal capacity in the world: China, Germany, Russia, the United Kingdom (UK), and the United States (US). Coal capacity in China has been more than the sum of the other four countries over many years, reaching 434.87GW after 2006. China caught up to the US in terms of plant capacity after 2013. Also, coal percentages in China (65%~ 75%) was significantly higher than the other four countries, which reflects the fundamental difference of energy matrices in different countries (Additional file 3: Table S2).

Table 1 displays the mean and 95% confidence intervals of all covariates during the three periods of 2000~2004, 2005~2010 and 2011~2016; note that these summaries are averaged over countries and time; obtained from empirical data without any distribution assumptions. From the first period to the last, average age-standardized incidence rates from lung cancer decreased by 46 (i.e., from 454 to 408) per hundred thousand (10%) in males but increased by 12 (i.e., from 143 to 155) per hundred thousand (8%) in females. Coal capacity increased from 16 GW to 23 GW. Smoking prevalence decreased by 9% in males and 11% in females, respectively.

**Table 1:** Basic characteristics of analyzed countries, 2000–2016

Year	2000~2004		2005~2010		2011~2016	
	Mean	(2.5th −97.5th quantile)	Mean	(2.5th −97.5th quantile)	Mean	(2.5th −97.5th quantile)
Lung cancer incidence ^a^						
Males	454	(61~942)	435	(70 ~ 877)	408	(69~817)
Females	144	(29~451)	151	(30~442)	155	(30~452)
Coal capacity ^b^	16,009	(0.60~218,341)	19,332	(0~ 322,042)	22,821	(6~211,854)
Smoking prevalence ^c^						
Males	32	(12~54)	30	(1~52)	29	(12~51)
Females	13	(1~31)	12	(1~30)	12	(1~28)
Traffic index ^c^	28	(5~59)	28	(6~58)	30	(7~65)
Industrialization index ^c^	18	(3~37)	17	(4~35)	16	(4~34)
GDP (PPP) ^d^	743	(9~4472)	911	(12~4898)	1113	(14~6922)
Total coal consumption ^e^	1	(0~8)	2	(0~12)	2	(0~13)
Population ^f^						
Males	327	(6~1441)	345	(6~1510)	367	(6~1571)
Females	322	(6~1480)	339	(6 ~ 1548)	361	(6~1606)

GDP (PPP): gross domestic product adjusted by (Purchasing Power Parity)

^a^Unit: case per hundred thousands

^b^ Unit: megawatts (MW)

^c^ Unit: %

^d^ Unit: Billion 2011 USD

^e^ Unit: Quadrillion British Thermal Unit (QBtu)

^f^ Unit: hundred thousands

**Fig. 2:**
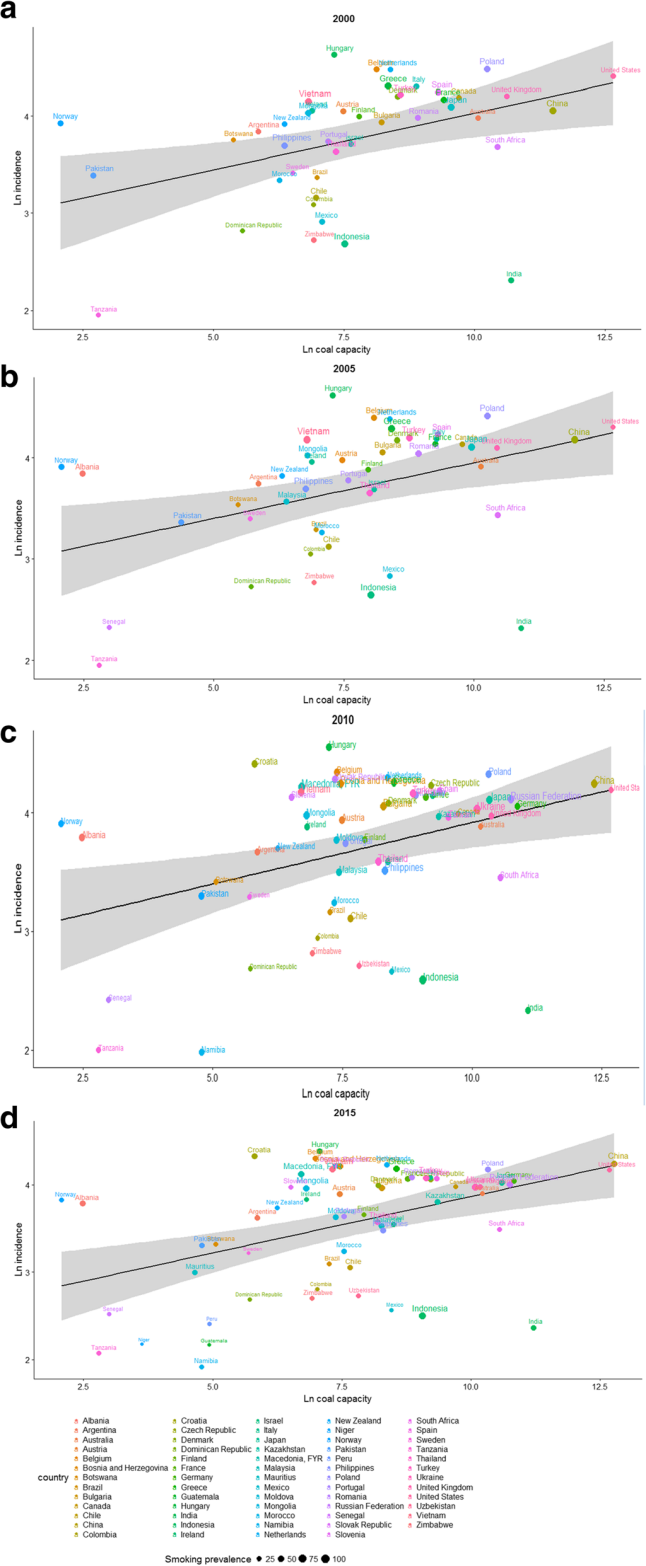
Incidence rates of lung cancer vs. coal capacity in (**a**) 2000; (**b**) 2005; (**c**) 2010 and (**d**) 2015 among males

**Fig. 3:**
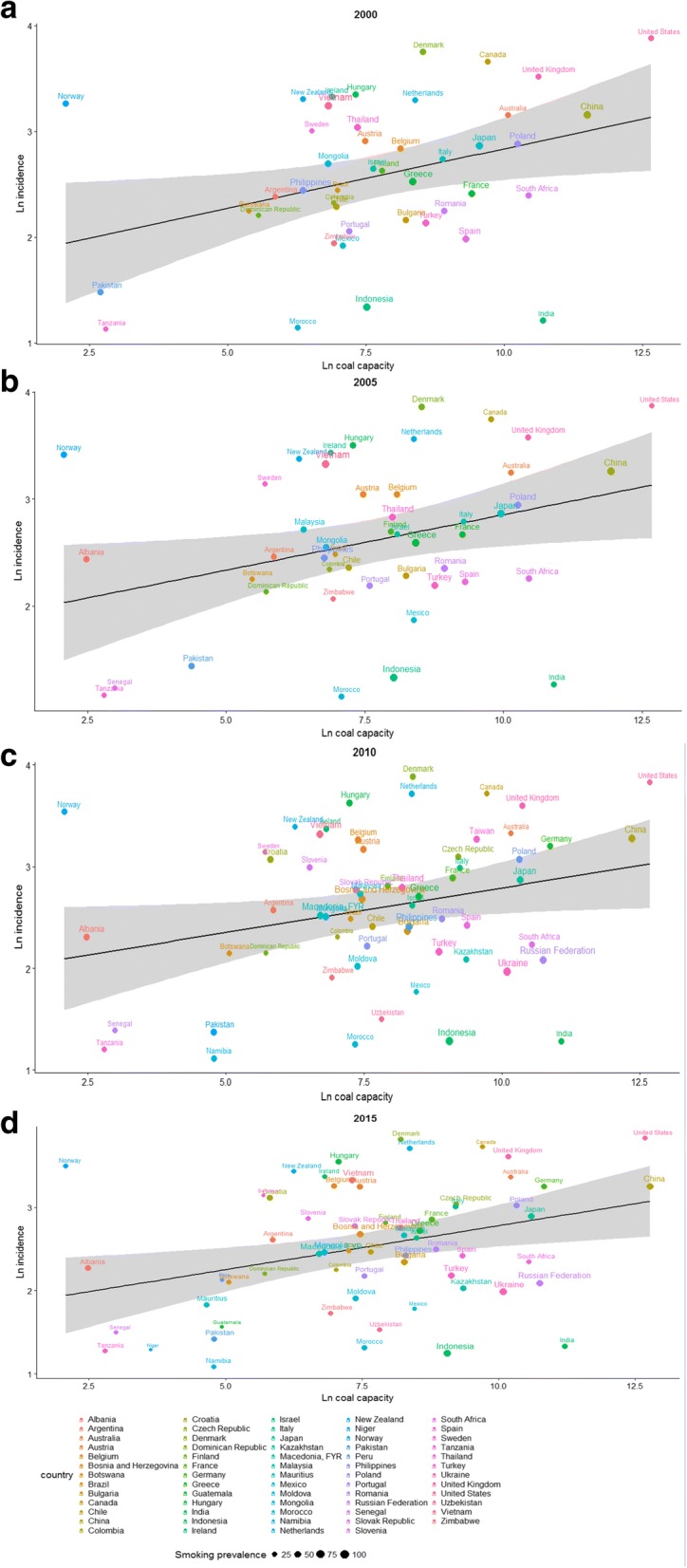
National incidence rates of lung cancer vs. coal capacity in (**a**) 2000; (**b**) 2005; (**c**) 2010 and (**d**) 2015 among females

Figure 2 (males) and Fig. 3 (females) show the relationship between 10-year-lag log coal capacity and log incidence rates of lung cancer in 2000, 2005, 2010 and 2015. Among both sexes, coal capacity was significantly positively correlated with lung cancer incidence rate (male, slopes = 0.10 to 0.13, all *p*-values < 0.05; females, slopes = 0.09 to 0.11, all p-values < 0.05).

Y axis: ln(lung cancer incidence rate), unit: ln(case/100 thousands); x axis: ln(*coal capacity*), unit: ln(MW); smoking prevalence: unit: %.

Y axis: ln(lung cancer incidence rate), unit: ln(case/100 thousands); x axis: ln(*coal capacity*), unit: ln(MW); smoking prevalence: unit: %.

Univariable, behavior-environmental, 5-year-lag, 10-year-lag and 15-year-lag models were applied to examine the effect among males and females, respectively (Table 2). Longer lag time of smoking of 20 and 30 years were also applied as sensitivity analysis (Additional file 1: Table S3). The point estimates of per capita coal capacity among the year-lag models were similar, so we picked the 10-year-lag model as our primary model. With a 1 KW increase of coal capacity per person in a country, the relative risk of lung cancer increases by a factor of 58.5% (95%CI = 7%~ 1.35%) among males and 85% (95%CI = 22%~ 182%) among females. Meanwhile, a 1% increase of smoking prevalence is associated with an increase of lung cancer incidence by a factor of 3% (95%CI = 1%~ 5%) and 2% (95%CI = 0%~ 5%), among males and females, respectively.

**Table 2:** Relative risk (RR) and 95% confidence intervals (CIs) of the increase in lung cancer incidence with change in coal capacity, among males and females

	Univariate		Behavior-Environmental		5-year-lag		10-year-lag		15-year-lag	
	RR	95% CI	RR	95% CI	RR	95% CI	RR	95%CI	RR	95% CI
Males										
Intercept	3.16 × 10^− 4^	(1.82 ~ 5.49) × 10^− 4^	7.83 × 10^− 5^	(0.29 ~ 2.09) × 10^− 4^	3.20 × 10^− 5^	(0.77 ~ 13.2) × 10^−5^	3.12 × 10^− 5^	(0.74 ~ 13.2) × 10^− 5^	2.82 × 10^− 5^	(0.60~13.3) × 10^− 5^
Per capita coal capacity ^a^	2.62	(1.40 ~ 4.90)	3.88	(2.22 ~ 6.78)	1.68	(1.10 ~ 2.56)	1.59	(1.07 ~ 2.35)	1.57	(1.05~2.35)
Smoking prevalence ^b^			1.03	(1.02 ~ 1.05)	1.03	(1.01 ~ 1.05)	1.03	(1.01 ~ 1.05)	1.03	(1.01~1.06)
Non-coal capacity ^a^					0.94	(0.79 ~ 1.12)	0.92	(0.76 ~ 1.10)	0.90	(0.73~1.11)
Traffic index ^b^					1.00	(0.98 ~ 1.02)	1.00	(0.98 ~ 1.02)	1.00	(0.98~1.03)
Industrialization index ^b^					1.03	(1.00 ~ 1.05)	1.03	(1.00 ~ 1.05)	1.03	(1.00~1.05)
GDP (PPP) per capita ^c^					1.00	(1.00 ~ 1.00)	1.00	(1.00 ~ 1.00)	1.00	(1.00~1.00)
Total coal consumption ^d^					1.01	(1.00 ~ 1.02)	1.01	(1.00 ~ 1.02)	1.01	(1.00~1.02)
QIC	−5,828,520		−5,812,294		− 5,134,366		−5,133,338		− 5,043,156	
Females										
Intercept	1.03 × 10^−4^	(0.58~ 1.86) × 10^− 4^	1.04 × 10^− 4^	(0.49 ~ 2.20) × 10^− 4^	1.21 × 10^−5^	(0.35 ~ 4.13) × 10^− 5^	1.16 × 10^− 5^	(0.34 ~ 3.99) × 10^− 5^	1.08 × 10^− 5^	(0.33~3.57) × 10^− 5^
Per capita coal capacity ^a^	3.87	(2.23 ~ 6.69)	3.95	(2.71 ~ 5.76)	1.84	(1.16 ~ 2.93)	1.85	(1.22 ~ 2.82)	1.85	(1.22~2.80)
Smoking prevalence ^b^			1.00	(0.96 ~ 1.04)	1.02	(1.00 ~ 1.05)	1.02	(1.00 ~ 1.05)	1.02	(1.00~1.05)
Non-coal capacity ^a^					1.00	(0.80 ~ 1.26)	0.99	(0.78 ~ 1.24)	0.98	(0.77~1.25)
Traffic index ^b^					1.00	(0.99 ~ 1.02)	1.00	(0.99 ~ 1.02)	1.00	(0.99~1.02)
Industrialization index ^b^					1.06	(1.023 ~ 1.09)	1.06	(1.03 ~ 1.10)	1.06	(1.03~1.10)
GDP (PPP) per capita ^c^					1.00	(1.00 ~ 1.00)	1.00	(1.00 ~ 1.00)	1.00	(1.00~1.00)
Total coal consumption ^d^					1.02	(1.00 ~ 1.04)	1.02	(1.01 ~ 1.04)	1.02	(1.01~1.04)
QIC	−1,623,308		−1,610,209		− 1,488,001		−1,488,392		− 1,459,133	

*RR* relative risk, *95% CI* 95% confidence interval, *GDP (PPP)* gross domestic product adjusted by (Purchasing Power Parity)

^a^ Unit: KW/capita

^b^ Unit: %

^c^ Unit: Year 2011 USD/capita

^d^ Unit: Quadrillion British Thermal Unit (QBtu

No statistically significant interactions between smoking and coal capacity, or any other time-varying effects on the estimates, were discovered, and thus these results were omitted. In the falsification test, coal capacity was not associated with CRC incidence rates in either males or females for any lag model (Additional file 1: Table S4).

Additional file 4: Table S5 presents the PAFs and standardized lung cancer cases attributable to coal-fired power plants among males and females, respectively, in 2015 and 2025. PAFs are higher for females than males in most countries due to higher RRs. Australia (39.26%) and US (32.65%) had the highest PAFs in 2015, corresponding to more than ten thousands and 233 thousands standardized lung cancer among females, respectively. In China, we estimated more than 347 thousand (range = 341,000~355,000) standardized lung cancer among females (PAF = 19%) and 786,000 (range = 769,000~803,000) among males (PAF = 15%) in 2025, based on different fertility scenarios estimated from UN.

## Discussion

Calculating per capita coal capacities as a determinant of lung cancer is a novel approach and should be interpreted differently from PM as seen in most studies. Firstly, per capita coal capacities could be regarded as averaged individual energy consumption from coal for every citizen within a country, thus may provide a meaningful approach to energy policy compared to PM. As countries compose their Intended Nationally Determined Contributions (INDC) goals for the coming decades, an analysis on reducing construction of or shutting down existing coal power plants may reveal further co-benefits of mitigating global warming and adverse health outcomes [33]. Secondly, since all pollutants related to lung cancer are not known, and known pollutants compose a small fraction of PM_2.5_, per capita coal capacity could serve as a better estimate of externality then pollutant composition measurements. Those pollutants such as SOx, NOx, heavy metal are associated with lung cancer from previous studies [34]. Thirdly, although capacity factors varied among countries, the range of capacity was approximately 40–60% [35]; this indicates that the quantity of coal combustion remained fixed after a plant was built. Finally, coal prices in a local market reflect coal quality. Although coal quality might vary between countries, it remains constant within a plant across time [36]. Country-specific effects, such as coal quality, are marginalized out by GEE in the analysis. By weighting the model by country population, we are reflecting the individual data by exploiting aggregated mean values of per capita coal capacity for each individual.

The association between per capita coal capacity and lung cancer incidence can be used to understand the potential number of lives affected by different levels of reliance on coal power. In 2015, we estimate a total of 865,805 male and 542,848 female standardized lung cancer cases can be attributed to anthropogenic power plants using coal as primary energy source. There is little difference between the lag 5 and lag 10 models in terms of quadratic information criterion (QIC) [37] and coefficients, and longer period of latency for smoking also yields similar results. Therefore, for sake of consistency with the other covariates, we fix lag 10 for coal capacity as primary model and estimate PAFs. These numbers should be interpreted as the total attributable cases given every country has WHO 2000–2025 standardized population and should not be compared directly to other estimations. However, these numbers adjust for age distributions in different countries and can be a valuable tool for country-to-country comparisons of the effect from coal capacity.

These estimates are comparable with prior reports but should be interpreted differently. The Global Burden of Disease group estimated that ambient air pollution globally caused 278.29 thousand lung cancer deaths for males in 2015 [38]. WHO suggested a total of three million deaths were attributable to ambient air pollution in 2012 based on PM2.5 measurement [39]. However, the above method barely linked to PM2.5 or its components. The Health and Environment Alliance estimated a total of 22,900 premature all-cause deaths due to coal-fired power plants in the EU in 2013 [40]. The study provides a direct approach for calculating health effects attributable to coal capacity at the national level.

The model also provides a hint of the effect sizes from coal fired power plant and smoking prevalence. Comparing 2005 to 2015 in U.S., 10-year-lag coal capacity increased from 321.06 GW to 322.29 GW, corresponding to an increase of 0.12 KW/person. Meanwhile, 10-year-lag smoking prevalence decreased 3.50% among males (data not shown). The increased per capita coal capacity is associated with the higher risk of lung cancer by a factor of 5.68% (=1.59^0.12^) while the decreasing smoking prevalence prevented the risk by a factor of 11.28% (=1.03^3.50^). This is meant as a quick numerical check; however, one should not try to surmise any statistical results from this.

## Study limitations

Despite using an ecological study design, biological plausibility of our results, the lack of any association in the falsification analysis, and the consistence of our estimates with those from previous investigations indicate that a strong impact of ecologic bias is very unlikely. [41] Moreover, our analysis on aggregated data is meant to infer policy decisions at the national level and for international comparison [42]. Other factors that may lead to overestimation or underestimation related to the ecological design should also be considered hereafter. To address concerns of data quality and other country-specific biases, we fitted a Poisson regression longitudinal model with GEE to account for time-independent confounders such as underreporting and/or over-diagnosis of diseases. GEE is a semiparametric technique in that it makes no assumptions about the correlation structure among outcomes. One disadvantage regarding GEE is potential efficiency losses compared to mixed models, if we could have correctly specified the true correlation structure properly in a parametric form. However, we are willing to sacrifice some efficiency for statistical robustness, a property GEE possesses while mixed models do not [43]. Regardless, this disadvantage would be germane had we failed to reject that coal capacity has null effect on lung cancer, but since we did reject, fitting with a correctly specified mixed model would only serve to increase the significance of the effect.

Our identified confounders associated with both coal capacity and lung cancer at the national level included adjustments for the appropriate latency period and strong temporality justifications for causal inference [44]. However, residual and unmeasured confounders, such as national-level educational attainment or occupational exposure, may exist; adding more parameters to our analysis would destabilize estimates and cause loss of statistical power. Potential misclassifications of meteorological factor such as wind directions, and/or geographical factors, cannot be adjusted in our model. Since neither the electricity matrix nor meteorological/geographical factor is relevant to a country’s healthcare system, misclassification is non-differential and more likely biases toward the null. Potential misclassifications of lung cancer diagnosis must also be considered across countries even GBD study is the best available data we can obtain [10]. The GBD study does not provide different types of lung cancer incidence for country-to-country comparison. Both adenocarcinoma [45] and squamous cell carcinoma [46, 47] of lung might have association with environmental factors. Further studies focusing on different types of cancer and coal-fired power plants should be conducted.

Our estimates may be conservative since not all time-varying covariates were considered in our model, such as indoor biomass combustion [48–51]. Although most countries included in this study were high-income countries and used a limited proportion of indoor biomass combustion, the true effect of coal power plants might be even higher if biomass combustion remained constant rather than decreasing. We adjusted total coal consumption in the model, which included the indoor combustion. Not considering control technologies in place of coal-fired power plants might lead to misclassification of the exposure level. Previous studies have showed that 10% national reduction on SOx emissions were associated with lower CVD incidence rates by 0.28% for males and 1.69% lower for females, respectively [52]. Further studies should address the effectiveness in terms of incidence from lung cancer. Finally, although smoking is unlikely to be a confounder at national level (due to lack of association with coal capacity), we are still interested in considering the nuanced differences of smoking prevalence and included in the model since it might be collinear with uncontrolled confounding from occupational exposures. The differences might exist among age, heavy or light smoking and/or synergistic effects between tobacco smoking and environmental exposure.

## Conclusion

We demonstrated an association between lung cancer incidence and coal-fired power plants via a novel approach that measures per capita coal capacity rather than PM. The study may be helpful in addressing a key policy question about the externality cost of coal power plants and estimates of the global disease burden from preventable lung cancer attributable to coal-fired power plants. Further studies might focus on the effectiveness of pollutant controls on health outcomes, quality of coal, synergistic effects between tobacco smoking and environmental exposure, and the financial burden of coal on healthcare expenditures.

## Additional files

Additional file 1: **Table S1**. Countries included in the analysis, by geographical region ^a^ (*N* = 83). **Table S3.** Relative risk (RR) and 95% confidence intervals (CIs) of the increase in lung cancer incidence with change in coal capacity, among males and females. **Table S4.** Relative risk (RR) and 95% confidence intervals (CIs) of the increase in colorectal cancer with change in coal capacity, adjusted for different variables in different models among males and females. (DOCX 23 kb)

Additional file 2: **Figure S1.** Coal capacity, plant capacity, coal percentage and total coal consumption of the top 5 countries with the highest levels of coal capacity in the world. (JPG 755 kb)

Additional file 3: **Table S2.** Estimated population attributable factors (2015, 2025) and standardized attributable cases (2015) among males and females of studied countries. (XLSX 40 kb)

Additional file 4: **Table S5.** National cancer incidence of lung, colorectum, population, smoking prevalence by gender and coal capacity in 2000, 2005, 2010, and 2015. (XLSX 44 kb)
