# Supplementary material for: A global perspective on coal-fired power plants and burden of lung cancer
Source: Environ Health. 2019 Jan 28;18:9. doi: 10.1186/s12940-019-0448-8 (PMC6350330; doi:10.1186/s12940-019-0448-8)
Supplement: Supplementary file 1 — Table S1. Countries included in the analysis, by geographical region a (N = 83). Table S3. Relative risk (RR) and 95% confidence intervals (CIs) of the increase in lung cancer incidence with change in coal capacity, among males and females. Table S4. Relative risk (RR) and 95% confidence intervals (CIs) of the increase in colorectal cancer with change in coal capacity, adjusted for different variables in different models among males and females. (DOCX 23 kb) [file 12940_2019_448_MOESM1_ESM.docx]

**Table 1. Basic characteristics of analyzed countries, 2000-2016**

| Year | 2000~2004 | | 2005~2010 | | 2011~2016 | |
| --- | --- | --- | --- | --- | --- | --- |
|  | Mean | (2.5^th^ -97.5^th^ quantile) | Mean | (2.5^th^ -97.5^th^ quantile) | Mean | (2.5^th^ -97.5^th^ quantile) |
| Lung cancer incidence ^a^ |  |  |  |  |  |  |
| Males | 454 | (61~942) | 435 | (70 ~877) | 408 | (69~817) |
| Females | 144 | (29~451) | 151 | (30~442) | 155 | (30~452) |
| Coal capacity ^b^ | 16009 | (0.60~218341) | 19332 | (0~ 322042) | 22821 | (6~211854) |
| Smoking prevalence ^c^ |  |  |  |  |  |  |
| Males | 32 | (12~54) | 30 | (1~52) | 29 | (12~51) |
| Females | 13 | (1~31) | 12 | (1~30) | 12 | (1~28) |
| Traffic index ^c^ | 28 | (5~59) | 28 | (6~58) | 30 | (7~65) |
| Industrialization index ^c^ | 18 | (3~37) | 17 | (4~35) | 16 | (4~34) |
| GDP (PPP) ^d^ | 743 | (9~4472) | 911 | (12~4898) | 1113 | (14~6922) |
| Total coal consumption ^e^ | 1 | (0~8) | 2 | (0~12) | 2 | (0~13) |
| Population ^f^ |  |  |  |  |  |  |
| Males | 327 | (6~1441) | 345 | (6~1510) | 367 | (6~1571) |
| Females | 322 | (6~1480) | 339 | (6 ~1548) | 361 | (6~1606) |

GDP (PPP): gross domestic product adjusted by (Purchasing Power Parity)

^a^ Unit: case per hundred thousands

^b^ Unit: megawatts (MW)

^c^ Unit: %

^d^ Unit: Billion 2011 USD

^e^ Unit: Quadrillion British Thermal Unit (QBtu)

^f^ Unit: hundred thousands

**Table 2. Relative risk (RR) and 95% confidence intervals (CIs) of the increase in lung cancer incidence with change in coal capacity, among males and females.**

|  | **Univariate** | | **Behavior-Environmental** | | **5-year-lag** | | **10-year-lag** | | **15-year-lag** | |
| --- | --- | --- | --- | --- | --- | --- | --- | --- | --- | --- |
| **Males** | RR | 95%CI | RR | 95%CI | RR | 95%CI | RR | 95%CI | RR | 95%CI |
| Intercept | 3.16×10­^-4^ | (1.82 ~ 5.49) ×10­^-4^ | 7.83×10­^-5^ | (0.29 ~ 2.09) ×10­^-4^ | 3.20×10­^-5^ | (0.77 ~ 13.2) ×10­^-5^ | 3.12×10­^-5^ | (0.74 ~ 13.2) ×10­^-5^ | 2.82×10­^-5^ | (0.60~13.3) ×10­^-5^ |
| Per capita coal capacity ^a^ | 2.62 | (1.40 ~ 4.90) | 3.88 | (2.22 ~ 6.78) | 1.68 | (1.10 ~ 2.56) | 1.59 | (1.07 ~ 2.35) | 1.57 | (1.05~2.35) |
| Smoking prevalence ^b^ |  |  | 1.03 | (1.02 ~ 1.05) | 1.03 | (1.01 ~ 1.05) | 1.03 | (1.01 ~ 1.05) | 1.03 | (1.01~1.06) |
| Non-coal capacity ^a^ |  |  |  |  | 0.94 | (0.79 ~ 1.12) | 0.92 | (0.76 ~ 1.10) | 0.90 | (0.73~1.11) |
| Traffic index ^b^ |  |  |  |  | 1.00 | (0.98 ~ 1.02) | 1.00 | (0.98 ~ 1.02) | 1.00 | (0.98~1.03) |
| Industrialization index ^b^ |  |  |  |  | 1.03 | (1.00 ~ 1.05) | 1.03 | (1.00 ~ 1.05) | 1.03 | (1.00~1.05) |
| GDP (PPP) per capita ^c^ |  |  |  |  | 1.00 | (1.00 ~ 1.00) | 1.00 | (1.00 ~ 1.00) | 1.00 | (1.00~1.00) |
| Total coal consumption ^d^ |  |  |  |  | 1.01 | (1.00 ~ 1.02) | 1.01 | (1.00 ~ 1.02) | 1.01 | (1.00~1.02) |
| QIC | -5828520 | | -5812294 | | -5134366 | | -5133338 | | -5043156 | |
| **Females** | RR | 95%CI | RR | 95%CI | RR | 95%CI | RR | 95%CI | RR | 95%CI |
| Intercept | 1.03×10­^-4^ | (0.58~ 1.86) ×10­^-4^ | 1.04×10­^-4^ | (0.49 ~ 2.20) ×10­^-4^ | 1.21×10­^-5^ | (0.35 ~ 4.13) ×10­^-5^ | 1.16×10­^-5^ | (0.34 ~ 3.99) ×10­^-5^ | 1.08×10­^-5^ | (0.33~3.57) ×10­^-5^ |
| Per capita coal capacity ^a^ | 3.87 | (2.23 ~ 6.69) | 3.95 | (2.71 ~ 5.76) | 1.84 | (1.16 ~ 2.93) | 1.85 | (1.22 ~ 2.82) | 1.85 | (1.22~2.80) |
| Smoking prevalence ^b^ |  |  | 1.00 | (0.96 ~ 1.04) | 1.02 | (1.00 ~ 1.05) | 1.02 | (1.00 ~ 1.05) | 1.02 | (1.00~1.05) |
| Non-coal capacity ^a^ |  |  |  |  | 1.00 | (0.80 ~ 1.26) | 0.99 | (0.78 ~ 1.24) | 0.98 | (0.77~1.25) |
| Traffic index ^b^ |  |  |  |  | 1.00 | (0.99 ~ 1.02) | 1.00 | (0.99 ~ 1.02) | 1.00 | (0.99~1.02) |
| Industrialization index ^b^ |  |  |  |  | 1.06 | (1.023 ~ 1.09) | 1.06 | (1.03 ~ 1.10) | 1.06 | (1.03~1.10) |
| GDP (PPP) per capita ^c^ |  |  |  |  | 1.00 | (1.00 ~ 1.00) | 1.00 | (1.00 ~ 1.00) | 1.00 | (1.00~1.00) |
| Total coal consumption ^d^ |  |  |  |  | 1.02 | (1.00 ~ 1.04) | 1.02 | (1.01 ~ 1.04) | 1.02 | (1.01~1.04) |
| QIC | -1623308 | | -1610209 | | -1488001 | | -1488392 | | -1459133 | |

RR: relative risk; 95%CI: 95% confidence interval; GDP (PPP): gross domestic product adjusted by (Purchasing Power Parity)

^a^ Unit: KW/capita

^b^ Unit: %

^c^ Unit: Year 2011 USD/capita

^d^ Unit: Quadrillion British Thermal Unit (QBtu)
