# Supplementary figures and images for: A global perspective on coal-fired power plants and burden of lung cancer
Source: Environ Health. 2019 Jan 28;18:9. doi: 10.1186/s12940-019-0448-8 (PMC6350330; doi:10.1186/s12940-019-0448-8)

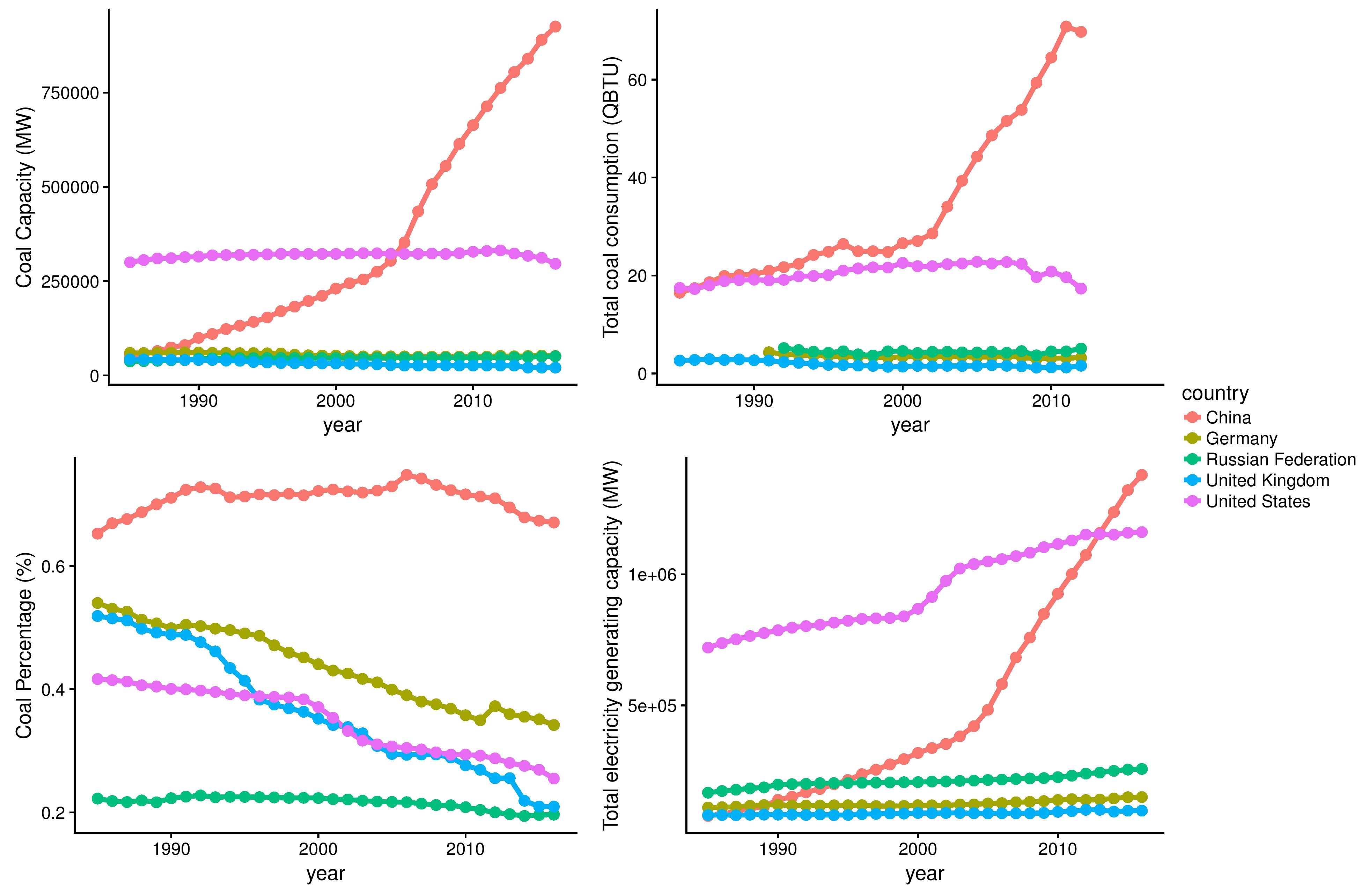

Supplement: Supplementary file 2 — Figure S1. Coal capacity, plant capacity, coal percentage and total coal consumption of the top 5 countries with the highest levels of coal capacity in the world. (JPG 755 kb) [file 12940_2019_448_MOESM2_ESM.jpg]
